# Viability and Functionality of Neonatal Porcine Islet-like Cell Clusters Bioprinted in Alginate-Based Bioinks

**DOI:** 10.3390/biomedicines10061420

**Published:** 2022-06-15

**Authors:** Sarah Duin, Shreya Bhandarkar, Susann Lehmann, Elisabeth Kemter, Eckhard Wolf, Michael Gelinsky, Barbara Ludwig, Anja Lode

**Affiliations:** 1 Centre for Translational Bone, Joint and Soft Tissue Research, University Hospital Carl Gustav Carus, Faculty of Medicine, Technische Universität, 01307 Dresden, Germany; shreyabhandarkar1997@gmail.com (S.B.); michael.gelinsky@tu-dresden.de (M.G.); anja.lode@tu-dresden.de (A.L.); 2 Paul Langerhans Institute Dresden of Helmholtz Centre Munich, University Hospital Carl Gustav Carus, Technische Universität Dresden, 01307 Dresden, Germany; susann.lehmann2@uniklinikum-dresden.de (S.L.); barbara.ludwig@uniklinikum-dresden.de (B.L.); 3 Paul Langerhans Institute Dresden of Helmholtz Centre Munich, German Centre for Diabetes Research (DZD), 01307 Dresden, Germany; 4 Gene Center and Center for Innovative Medical Models (CiMM), Molecular Animal Breeding and Biotechnology, Ludwig-Maximilians University Munich, 81377 München, Germany; kemter@genzentrum.lmu.de (E.K.); ewolf@genzentrum.lmu.de (E.W.); 5 German Centre for Diabetes Research (DZD), 85764 Neuherberg, Germany; 6 Medical Clinic III, University Hospital Carl Gustav Carus, Technische Universität, 01307 Dresden, Germany

**Keywords:** 3D bioprinting, alginate-methylcellulose, neonatal porcine islet-like cell clusters, functionality, glucose-responsiveness

## Abstract

The transplantation of pancreatic islets can prevent severe long-term complications in diabetes mellitus type 1 patients. With respect to a shortage of donor organs, the transplantation of xenogeneic islets is highly attractive. To avoid rejection, islets can be encapsulated in immuno-protective hydrogel-macrocapsules, whereby 3D bioprinted structures with macropores allow for a high surface-to-volume ratio and reduced diffusion distances. In the present study, we applied 3D bioprinting to encapsulate the potentially clinically applicable neonatal porcine islet-like cell clusters (NICC) in alginate-methylcellulose. The material was additionally supplemented with bovine serum albumin or the human blood plasma derivatives platelet lysate and fresh frozen plasma. NICC were analysed for viability, proliferation, the presence of hormones, and the release of insulin in reaction to glucose stimulation. Bioprinted NICC are homogeneously distributed, remain morphologically intact, and show a comparable viability and proliferation to control NICC. The number of insulin-positive cells is comparable between the groups and over time. The amount of insulin release increases over time and is released in response to glucose stimulation over 4 weeks. In summary, we show the successful bioprinting of NICC and could demonstrate functionality over the long-term in vitro. Supplementation resulted in a trend for higher viability, but no additional benefit on functionality was observed.

## 1. Introduction

Type 1 diabetes mellitus (T1D) is a mostly immune-mediated disease characterised by the destruction of pancreatic β-cells, which results in an almost complete lack of insulin in the body. With clinical onset of the disease, the patients become dependent on exogenous replacements of insulin via regular injections. Even with supreme management of the injections, it remains impossible to achieve comparable metabolic control to endogenous insulin-secretion [1], resulting in hyperglycaemia-induced long-term complications such as nephropathy or cardiovascular disease and a reduced life-expectancy [2,3]. Endogenous insulin-secretion can at least be partially restored by the transplantation of pancreatic islets of Langerhans containing the insulin-producing β-cells, whereby the gold standard is infusion of pancreatic islets into the portal vein [4]. Mainly due to early hypoxia, coagulation, and an instant blood-mediated inflammatory reaction (IBMIR), a considerable number of islets are lost shortly after intraportal transplantation [5,6]. Thus, in many cases islets from two donors are required to achieve euglycaemia in the recipient [7]. Coupled with the general shortage of donor organs, the necessity for life-long immune-suppression and a progressive loss of function [8], islet transplantation is only performed in carefully selected, very severe cases of T1D [9,10]. Nevertheless, even if the transplant progressively loses function, the patients experience a long-term clinical benefit of improved glycaemic control and fewer complications [4,6], but it is impossible to extend this therapeutic option to a wider range of patients without an alternative islet source.

A potentially unlimited source of insulin-producing cells are xenogeneic islets. Hereby, a realistic and much-researched donor species for human beings is the domestic pig [11], as porcine islets are easy to isolate in reproducible quality [12] and cover a comparable range of metabolic fluctuations to human islets [13]. In contrast to adult porcine islets, which show a high fragility and a low viability after isolation, neonatal porcine pancreatic islet-like cell clusters (NICC) are easy to isolate in a reproducibly good quality with clinical-grade purity, remain stable and show a higher viability in vitro, and display an increased resistance towards hypoxia-induced apoptosis [7,12,14], making them potentially relevant for clinical transplantation. However, compared to adult islets, NICC, which are derived from an immature pancreas of neonatal piglets, contain a comparatively low number of β-cells, and need to mature for 6–8 weeks either in vitro or in vivo before showing an adequate metabolic reaction and normalising blood-glucose levels [7,14,15,16,17,18].

The current state-of-the-art transplantation mode of islet allotransplantation is the intraportal infusion of free islets into the portal vein of the recipient’s liver. As the liver transplant site exhibits some hurdles for engraftment and long-term graft function such as the occurrence of IBMIR or the low oxygen content in portal vein blood, alternative transplant sites for free islet transplantation are currently under investigation [19,20]. Using a xenograft for free islet transplantation, the graft rejection mechanisms could be more severe compared to the allotransplantation setting, due to more immunological differences between donor and recipient. Both in free islet allo- and xenotransplantation settings, a continuous immunosuppressive regime is required to protect the graft from immune attack.

Currently, the most promising strategy to circumvent (heavy) immunosuppression is the encapsulation of insulin-producing cells, i.e., the use of a semi-permeable physical barrier between the transplanted cells and the host’s immune system. Encapsulation strategies vary between microencapsulation (small groups of generally 1–3 islets encased separately) and macroencapsulation (a large number of islets collectively encased in a macroscopic device). Macroencapsulation devices can be intravascular, i.e., directly connected to the vasculature, which allows for a rapid exchange of glucose and insulin but comes at a high risk for thrombosis; or can be extravascular, generally implanted subcutaneously or intraperitoneally [21]. Microencapsulation strategies were able to restore normoglycaemia without immunosuppression in rodents in a number of studies, but results in large animals and humans varied strongly (reviewed in [22]).

Due to this difficulty in the transfer of microcapsules to larger animals, and the risk of thrombosis in case of intravascular macrocapsules, extravascular macroencapsulation strategies, often based on alginate, are currently regarded as the most promising option as summarised in a recent review [23]. In general, macroencapsulation approaches have a number of advantages for the transplantation of pancreatic islets, such as the possibility for a high islet density and easy retrievability. Furthermore, porcine islets encased in an alginate macrocapsule have already been shown to restore normoglycaemia in diabetic primates for up to 6 months before graft failure, whereby failure was attributed to the lifespan of adult porcine islets [24]. Nevertheless, the upscaling to clinically relevant sizes, and especially to clinically relevant islet densities, remains a major challenge, mainly due to increasing diffusion distances [25,26].

The development of macrocapsules with defined macropores to increase the surface-to-volume ratio therefore seems to be a promising strategy, which can be realised by employing 3D bioprinting. 3D bioprinting refers to using additive manufacturing methods with cell-containing hydrogels (bioinks). Hereby, the most prominent are extrusion-based methods, in the present case using pneumatic pressure to deposit strands of a material in a layer-by-layer fashion. With such additive manufacturing technologies, it is possible to generate constructs with defined pore architecture (hereafter referred to as ‘scaffolds’) in clinically relevant sizes [27]. Yet, despite the advantages of direct cell inclusion and short diffusion distances, up to now only few studies on 3D bioprinting of pancreatic islets have been published. Bioprinting of islets is a complex issue, as in addition to the requirements for normal encapsulation, i.e., immune protection and support of functionality, it is also required that the inks are viscous enough to remain stable until crosslinking. To achieve this, oftentimes the biopolymer concentration is strongly increased in comparison to low-viscous gels such as plain alginate, which can result in shear stress on the cells and can interfere with the diffusion of glucose and insulin. Pancreatic islets are cell clusters of up to 300 µm in diameter, and a large concern in handling islets is damage through shear stress. The shear stress during the isolation can lead to some fragmentation [28] and shear damage has been reported to decrease the functionality of pancreatic islets [29]. For the bioprinting of islets, it has been reported that pressures of 30 kPa and higher during printing negatively impacted islet viability of different species and led to a size reduction of adult porcine islets when incorporated into 3% plain alginate and bioprinted with a nozzle diameter of 580 µm [30]. In contrast to this, for adult murine islets incorporated into the much more viscous algMC and bioprinted with 30–50 kPa through an 840 µm nozzle, we did not observe an impact on viability in our previous work [31]. This is also reflected in the fact that in the majority of studies concerning extrusion-based bioprinting of islets known to the authors, the islets showed a high viability. Functionality on the other hand, could either not be achieved [32,33], or was not demonstrated in bioprinted scaffolds of a promising material [34]. In our previous work [31] we could show that after bioprinting adult murine islets using a bioink of clinically approved alginate and methylcellulose (algMC), they showed a viability comparable to free control islets, retained their morphology, and released insulin in a glucose-dependent fashion for up to 7 days after bioprinting. As expected for adult islets [35,36,37], functionality was reduced over time and lost after 10–14 days (unpublished data). In a more recent work, Idaszek et al. were able to show functionality of bioprinted adult porcine islets 24 h after bioprinting; however, functionality was lost after one additional day in culture [38]. To the best of the authors’ knowledge, no studies on bioprinting of NICC have been published as of now.

In the present work, we aimed at combining NICC, as an islet source with a high potential for xenotransplantation in clinical applications, with extrusion-based bioprinting, as a highly promising technology for the generation of scaffolds that allow for macroencapsulation while retaining short diffusion distances. Based on our proof-of-concept for the bioprinting of functional adult murine pancreatic islets, we used algMC for the bioprinting of NICC in the present study. Additionally, algMC was supplemented with different proteins and growth factors, specifically bovine serum albumin (BSA), human platelet lysate (PL), and human fresh frozen plasma (FFP). The choice of supplements is based on the observation of an increase in viability and insulin secretion when murine islets were exposed to human serum albumin [39], and an improved blood-glucose control after injections of platelet-rich plasma into diabetic rats [40,41].

## 2. Materials and Methods

### 2.1. Isolation and Culture of NICC

NICC were isolated at the Centre for Innovative Medical models (CiMM) in Munich (Germany) and transferred to Dresden via courier on day 3 after isolation. For isolation, pancreata were obtained from neonatal wild-type pigs <8 days of age and cut into small pieces with scissors. The pieces were digested with collagenase at 37 °C as described by Korbutt et al. [12] and the digest was filtered and washed repeatedly with Hank’s Balanced Salt Solution (HBSS) containing BSA. NICC were cultured for 3 days in recovery medium consisting of Ham’s F-12/M199 supplemented with protease inhibitors, antioxidants, and additional nutrients [42] at the CiMM before being shipped to Dresden in this medium on the third day of culture. Directly after arrival, medium was changed to maturation medium. Prepared as described by Korbutt et al. [12] this consisted of Ham’s F 10 (Sigma, St. Louis, MO, USA) supplemented with 0.5% (*w*/*v*) BSA (Roth, Karlsruhe, Germany), 10 mM glucose (Sigma), 50 µM 3-Isobutyl-1-methylxanthine (IBMX; Sigma), 100 U/mL penicillin, 100 μg/mL streptomycin, 2 mM L-glutamine, 10 mM nicotinamide (Sigma), and 1.6 mM CaCl_2_ (Merck Millipore, Darmstadt, Germany). For the first week of culture, the maturation medium was additionally supplemented with 50 nM retinoic acid (Sigma) and 1 µM triiodothyronine (T3; Sigma). NICC were cultured at 37 °C and 5% CO_2_ in suspension culture or in bioprinted scaffolds for up to 28 days with media changes every 2–3 days.

### 2.2. Preparation and Bioprinting of the Bioinks

All inks tested within this study were based on 3% (*w*/*v*) alginate solutions mixed with 9% (*w*/*v*) methylcellulose (MC) used as a thickening agent as first published by Schütz et al. and further characterised by Hodder et al. [27,43]. The basic algMC bioink consisted of clinical-grade sodium alginate (Pronova Up MVM, Novamatrix, Sandvika, Norway), chosen in regard to its use in islet transplantation studies [44]. Prior to gel preparation, alginate and MC (4000 cP; Sigma) powder had been sterilised by autoclaving. Directly before bioprinting, alginate was dissolved in phosphate buffered saline (PBS, Gibco^®^ by Life Technologies, Paisley, UK), 1% BSA (*w*/*v*) in PBS, PL, or FFP.

PL was prepared from five expired concentrates (i.e., 20 individual donors) of PRP obtained from the German Red Cross (BSD Ost, Dresden, Germany). Platelets were lysed via four consecutive freeze–thaw cycles (−80 °C and 37 °C) and pooled. After removal of cellular debris by centrifugation at 17,500× *g* for 20 min, the supernatant was further purified and sterilised by stepwise filtration through 5 µm, 1.2 µm cellulose acetate, and 0.2 µm polytetrafluoroethylene sterile filters, all with very low protein binding (Sarstedt, Nümbrecht, Germany). Until use for preparation of inks, PL was stored at −20 °C. FFP was acquired from German Red Cross (BSD Ost, Dresden, Germany). To minimize the influence of donor variations, plasma of 5 different donors was pooled for all the experiments presented in this study.

After complete dissolution of alginate through repeated stirring during a swelling time of 15–20 min, MC powder was added and left to swell for at least 30 min with repeated stirring before addition of NICC. For each experiment, NICC from 1–3 neonatal pigs were pooled on day 7 after isolation and up to 20,000 islet equivalents (IEQ) per gram ink were suspended in 150–300 µL maturation medium per gram, followed by careful incorporation into the material with a spatula.

The extrusion-based system used for 3D bioprinting was the BioScaffolder 3.1 (GeSiM mbH, Radeberg, Germany) operated under sterile conditions. The bioinks were carefully transferred into a sterile cartridge and dispensed through a dosing needle (Nordson EFD, Oberhaching, Germany), with an inner diameter of 840 µm, using pressures between 20–50 kPa. 3D scaffolds were constructed through deposition of a single meandering hydrogel strand per layer at a speed of 10 mm/s with a 90° change of orientation after each layer. Total scaffold dimensions were 4.5 mm diameter edge length, 3 mm strand distance, and 3 layers resulting in a total height of approximately 2 mm. After bioprinting, the scaffolds were crosslinked with 1 mL of 70 mM strontium chloride (SrCl_2_, Roth) solution for 10 min and kept under cell culture conditions.

### 2.3. Characterisation of Cell-Laden Scaffolds

#### 2.3.1. MTT and DTZ Staining

For a visual assessment of metabolic activity, samples were incubated in 0.5 mg/mL thiazolyl blue tetrazolium bromide (MTT, Sigma) in maturation medium under cell culture conditions for 2 h. For a visual assessment of presence of insulin, samples were briefly incubated in 2 mg/mL dithizone (DTZ, Sigma) dissolved in 20% dimethyl sulfoxide (DMSO, Sigma) in PBS. Images were taken using a stereo light microscope (LeicaM205 C, Leica, Wetzlar, Germany).

#### 2.3.2. Live/Dead Staining

Cell viability was determined using a Live/Dead viability/cytotoxicity kit (Molecular Probes, Eugene, OR, USA), containing calcein AM and ethidium homodimer-1 for the staining of live and dead cells, respectively, according to the manufacturer’s instructions. Imaging was performed using a fluorescence microscope Bz-X800 (Keyence, Osaka, Japan). For a semi-quantitative assessment of islet viability, bioprinted and free control NICC were imaged and visually sorted into viability categories (0, 25, 50, 75, 100% viable); % viability was calculated as described in Karaoz et al. [45].

#### 2.3.3. Immunofluorescence Staining

Bioprinted scaffolds containing NICC as well as control NICC in suspension culture were fixed overnight in formaldehyde (Merck Millipore) diluted to 4% (*v*/*v*) in HBSS (Thermo Fisher, Waltham, MA, USA) at 4 °C and washed with HBSS. All samples were embedded in Tissue-Tek O.C.T (Sakura Finetek, Torrance, CA, USA), a Microm HM 560 Cryostat (Thermo Fisher) was used to prepare cryosections. For immunostaining, the cryosections were incubated in PBS at 70 °C for 20 min, and permeabilized with 0.2% (*v*/*v*) Triton X-100 (Serva, Heidelberg, Germany) in PBS, whereby permeabilization was performed in three steps of 5 min incubation each. This was followed by incubation with background sniper (BS966L, Biocare medical, Pacheco, CA, USA) for 11 min at room temperature to block unspecific antibody binding sites. Cellbrite (Biotium, Fremont, CA, USA) was used for membrane staining prior to antibody staining.

Primary antibodies used were mouse monoclonal anti-insulin (clone K36AC10, 1:1000, I2018, Sigma), and rabbit polyclonal anti-glucagon (1:200, 2760S, Cell Signaling Technology, Danvers, MA, USA), applied overnight at 4 °C. Secondary antibodies used were goat anti-mouse Alexa Fluor 488 (1:500, A11001, Life Technologies), and goat anti-rabbit Alexa Fluor 568 (1:1000, A11011, Life Technologies), applied for 30 min at room temperature. Nuclei were stained with DAPI (5 µg/mL; Roche, Basel, Switzerland) for 1 h. To achieve the desired concentration, antibodies were diluted in blocking buffer (0.2% (*v*/*v*) Triton X-100, 2% (*w*/*v*) BSA and 2% (*v*/*v*) goat serum (Gibco) in PBS). All staining steps were alternated with at least one washing step with 0.1% PBS-Tween (Serva).

Cryosections were imaged on a confocal laser scanning microscope (cLSM) using a Leica TCS SP5 (Leica) located in the Core Facility Cellular Imaging (CFCI) of the Faculty of Medicine of TU Dresden.

#### 2.3.4. Staining for Apoptotic and Proliferating Nuclei

Apoptotic nuclei were stained with the In Situ Cell Death Detection Kit TMR red (Sigma), which detects TdT-mediated dUTP-X nick end labelling (TUNEL), according to the manufacturer’s instructions. TUNEL-staining was performed on cryosections. Apoptosis in islets was quantitatively assessed by counting nuclei of 25 islets of varying sizes and calculating % nuclei stained for TUNEL compared to all nuclei stained by DAPI.

For the detection of newly formed cells, the Click-iT EdU imaging kit (Thermo Fisher) was used. Full scaffolds containing NICC and free control NICC were incubated with 10 µM EdU in maturation medium for 24 h under cell culture conditions before fixation overnight in 4% formaldehyde in HBSS at 4 °C. Samples were then washed with HBSS and permeabilized with 0.5% Triton X-100 for 1 h. Detection of EdU was performed by preparing the reaction cocktail according to manufacturer’s instructions and incubating the samples for 1 h. Counterstaining of non-proliferating nuclei was performed using Hoechst 33342 at a concentration of 2 µg/mL.

All samples were imaged on a cLSM Leica TCS SP5 and image analysis was performed using Image J V1.53i (National Institutes of Health, Bethesda, MD, USA).

#### 2.3.5. Quantification of DNA

To determine the DNA-content, samples were stored at −20 °C until analysis. For scaffold dissolution, all samples were thawed and incubated in 100 mM sodium citrate on a shaker for 2–3 h. Free control NICC were treated identically. To lyse the cells, samples we incubated overnight in a 60 °C water bath followed by 10 min ultrasonication in an ice-cold water bath. The DNA-content in the lysates was measured using the QuantiFluor dsDNA system (Promega, Madison, WI, USA) as per the manufacturer’s instructions on a microplate reader (Infinite M200pro; Tecan, Männedorf, Switzerland) at excitation and emission wavelengths of 485 and 535 nm, respectively.

#### 2.3.6. Functional Analysis of Islets: Glucose Stimulated Insulin Release (GSIR)

Analysis of islet reaction to stimulation with glucose was done via GSIR assay. For the bio-printed NICC, whole scaffolds were used as single samples; for the control, each sample consisted of 50–100 NICC of varying sizes that had been picked manually. On day 1, 7, 14, 21, and 28 after bioprinting, samples were treated with low (3.3 mM) or high (16.4 mM) glucose in Krebs Ringer bicarbonate buffer (137 mM NaCl, 4.7 mM KCl, 1.2 mM KH_2_PO_4_, 1.2 mM MgSO_4_, 2.5 mM CaCl_2_, 25 mM NaHCO_3_ (all from Merck Millipore), 0.25% (*w*/*v*) BSA), and secreted insulin was quantified in the supernatant. The stimulation scheme was as follows: all samples were exposed to 3.3 mM glucose for 2 h (resting conditions) followed by stimulation with first low and then high glucose for 3 h each. During high-glucose stimulation, NICC were additionally exposed to 100 µM of the GLP-1 analogue liraglutide (Victoza^®^, Novo Nordisk A/S, Bagsværd, Denmark) per ml buffer solution.

Insulin was measured from supernatants stored at −20 °C until quantification via porcine insulin ELISA kits (Mercodia, Uppsala, Sweden) performed to the manufacturer’s instructions and assayed on a microplate reader (Infinite M200pro) at an absorbance of 450 nm. To obtain the stimulation index (SI), the ratio of insulin released in high versus low glucose conditions was calculated.

### 2.4. Statistics

Sample size is denoted by “*n* =” and expresses number of replicate experiments, always further detailed by the number of islets analysed. Each replicate experiment is based on pooled NICC from 1–3 piglets from a separate isolation. Data were tested for statistically significant differences (*p* < 0.05) using a 95% confidence interval. Two sample groups over multiple time points were compared via one-way ANOVA with post-hoc Sidak and multiple sample groups over multiple time points with two-way ANOVA with post-hoc Tukey. All statistical analyses were performed using GraphPad Prism 8 for Windows (GraphPad Software, San Diego, CA, USA).

## 3. Results

### 3.1. Distribution and Viability of NICC Bioprinted in algMC

As a first step in establishing bioprinting of potentially clinically applicable islets, we investigated the general feasibility of bioprinting viable NICC. The bioink chosen was the algMC blend that had previously been shown to be suitable for the embedding of adult murine islets via extrusion-based bioprinting [31].

**Figure 1:**
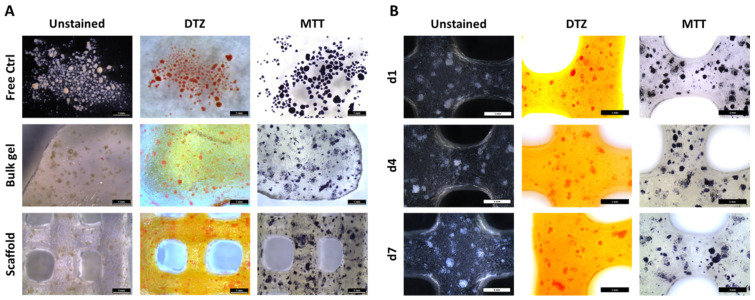
Distribution, insulin content (DTZ), and metabolic activity (MTT) of bioprinted NICC. (**A**) Influence of incorporation into algMC and bioprinting, d3 after incorporation. Free control NICC in suspension culture served as control for the influence of the material, unprinted NICC in algMC bulk gels as control for the extrusion process; (**B**) NICC in bioprinted scaffolds over 7 days of culture. Scale bars = 1 mm for all.

To get a visual overview over the morphology, distribution, metabolic activity, and presence of insulin, up to 20,000 IEQ were incorporated per gram of ink and NICC were stained with DTZ, a zinc-binding dye for the detection of insulin, and MTT, for visualizing metabolic activity. NICC in suspension culture served as a control for the influence of the material, unprinted NICC in algMC bulk gels as a control for the extrusion process (Figure 1A). NICC were homogeneously distributed inside the bulk gel as well as the bioprinted scaffolds. The presence of insulin and the metabolic activity could be detected in all analysed samples. The intensity of the stainings was comparable between the conditions (Figure 1A), i.e., NICC in suspension culture (“Free Ctrl”) and NICC in algMC, and the time points (Figure 1B) analysed. This indicates that the insulin content and metabolic activity were unaffected by the incorporation of NICC into algMC, and extrusion through the needle during printing, and also remained approximately constant over a culture period of 7 days. All bioprinted scaffolds remained stable and easy to handle during this incubation in maturation medium.

**Figure 2:**
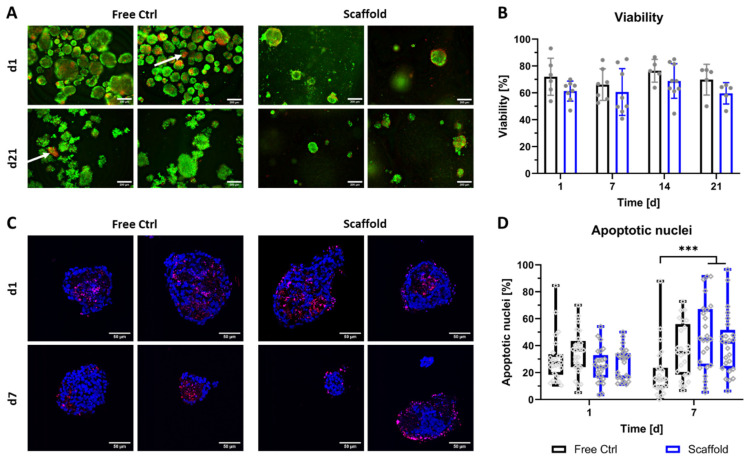
Viability of bioprinted NICC. (**A**) Live/dead staining of free control and bioprinted (“Scaffold”) NICC, representative images taken on d1 and d21 after printing, scale bars = 200 µm. White arrows indicate single islets with a majority of dead cells; (**B**) Semi-quantitative assessment of islet viability on the basis of live/dead stainings as shown in A. Mean ± SD, *n* ≥ 5, >25 NICC each. Repeat experiments are depicted as grey dots. Due to a limited number of samples, not all time points and conditions were analysed in all repeat experiments, which resulted in an *n* = 5–7 for free control, and an *n* = 5–9 for bioprinted scaffolds; (**C**) TUNEL staining for apoptotic nuclei in free control and bioprinted (“Scaffold”) NICC, representative images of samples fixed on d1 and d7 after printing, scale bars = 50 µm; (**D**) Quantitative analysis of percent apoptotic nuclei per islet. Mean ± SD, *n* = 2, 25 NICC each, *** *p* < 0.001. Repeat experiments depicted as neighbouring box plots, grey dots indicate individual islets.

Following investigation of the general compatibility of NICC with the algMC bioink, the viability of bioprinted NICC was examined in-depth via qualitative and quantitative analysis of staining for live vs. dead cells (Figure 2A,B) as well as staining for apoptotic nuclei (Figure 2C,D). Viability via staining for live vs. dead cells was examined in ≥5 repeat experiments for up to 21 days. Independent of the condition (free control vs. bioprinted scaffold) and time point, most NICC contained a majority of live (green) cells, and a limited number of dead (red) cells distributed over the single islet-like clusters. However, in the free control, a small but noticeable number of NICC consisted of a majority of dead cells (indicated by white arrows in Figure 2A), which was not observed in bioprinted NICC. The semi-quantitative evaluation of live/dead stainings revealed that the viability lay between 50 and 90% at all time-points with a large variability between the experiments. The viability of bioprinted NICC was slightly but not significantly lower than that of free control NICC throughout the entire time of observation.

The viability of the free control and bioprinted NICC was additionally examined via TUNEL staining for apoptotic nuclei in two repeat experiments for up to 7 days. In qualitative analysis, it was apparent that in both conditions the vast majority of islets retained their spherical morphology. Independent of the condition, in most NICC, apoptotic nuclei were either distributed throughout the whole islet cluster or located in the centre forming an apoptotic core. In a low number of bioprinted NICC, apoptotic cells were located preferentially at the outer border, which was not observed in the free control (Figure 2C). Independent of condition and time-point, many NICC displayed empty areas, which are within the boundaries of the islets, but contain neither DAPI- nor TUNEL-stained nuclei, indicating prior complete cell death. In quantitative analysis (Figure 2D), this is reflected as an average of 20–40% apoptotic nuclei. On day 1 after bioprinting, this range was highly similar between both groups and the repeat experiments. As the percentage of apoptotic cells decreased slightly in the free control in one of the experiments and that in the bioprinted samples increased slightly, this resulted in a significant difference between the groups on day 7. Free control as well as bioprinted NICC were present in a wide variety of sizes, whereby printed NICC were significantly smaller with a mean of 100 nuclei compared to free control NICC with a mean of 150 nuclei. Furthermore, in the free control some NICC contained as many as 500 nuclei, whereas the largest bioprinted NICC consisted of less than 400 nuclei (Figure S1).

### 3.2. Distribution, Viability, and Proliferation of NICC Bioprinted in Supplemented algMC

Having ascertained the general feasibility of bioprinting of NICC, with a mean viability of 60–70%, we further investigated the effect of the addition of supplementation with proteins or protein-growth factor mixtures to algMC on bioprinted NICC.

**Figure 3:**
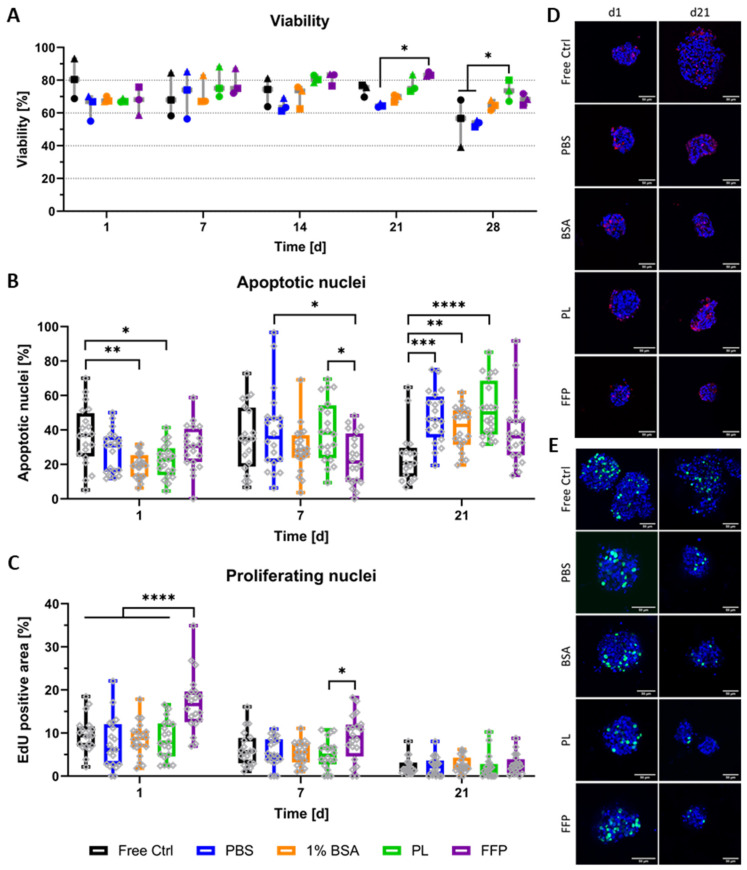
Viability and proliferation of bioprinted NICC in supplemented algMC. Analysed groups are free control NICC in suspension culture and NICC bioprinted in PBS-algMC, BSA-algMC, PL-algMC, and FFP-algMC. (**A**) Semi-quantitative analysis of live/dead staining, the different symbols represent the different experiments depicted, mean ± SD, *n* = 3, >25 NICC each; (**B**) Quantitative analysis of percent apoptotic nuclei per islet, mean ± SD, *n* = 1, 20 NICC; (**C**) Quantitative analysis of proliferating nuclei. Mean area of proliferating nuclei in percent of total islet area, mean ± SD, *n* = 1, 20 NICC; (**D**) TUNEL staining for apoptotic nuclei in bioprinted NICC, representative images of samples fixed on d1 and d21 after bioprinting, scale bars = 50 µm; (**E**) EdU staining for nuclei of cells that proliferated within 24 h before fixation, representative images of samples fixed on d1 and d21 after bioprinting, scale bars = 50 µm. Grey dots in a-c indicate individual islets, significances indicate * *p* < 0.05, ** *p* < 0.01, *** *p* < 0.001, **** *p* < 0.0001.

Supplements were added via the dissolution of alginate in 1% BSA, PL, or FFP. Accordingly, the different bioinks are designated as PBS-algMC (non-supplemented), BSA-algMC, PL-algMC, and FFP-algMC. NICC in the supplemented bioinks were compared to non-supplemented algMC and free control NICC in suspension culture (Figure 3). Similar to NICC in non-supplemented PBS-algMC, the distribution of NICC in bioprinted scaffolds was adequately homogenous, and the presence of insulin and metabolic activity were comparable between all bioinks tested. Furthermore, all bioprinted scaffolds remained stable over the entire culture time of 4 weeks, which is exemplarily depicted for scaffolds after 3 weeks of culture (Figure S2). Overall, the strands are slightly larger than the nozzle diameter due to some swelling during crosslinking, but qualitatively no further changes in appearance were visible over the entire culture period. Furthermore, all scaffolds retained their shape and integrity also during handling.

Semi-quantitative analysis from live/dead stainings (Figure 3A) was performed for three separate repeat experiments (i.e., NICC from three separate isolations), the separate experiments are depicted by different symbols. In general, viability ranged between 60 and 90% over the entire duration of observation and was comparable between all analysed groups. Especially at early time points, differences between the experiments were greater than those between the different conditions, whereas from day 14 onwards, values were predominantly similar between the different experiments and slight differences between the bioinks became apparent. Though in most cases not significant, by trend viability in supplemented bioinks was increased compared to PBS-algMC from day 14 onwards.

Additionally to live/dead stainings, NICC were analysed for apoptotic nuclei (Figure 3B,D) and islet size (Figure S3) in one of the repeat experiments, whereby this repeat experiment is depicted as dots in Figure 3A. Analogous to previous results (Figure 2), the staining for apoptotic nuclei on the cryosections depicted that the morphology of NICC was generally preserved and apoptotic nuclei were mostly distributed throughout the whole islet cluster (Figure 3D) but sporadic apoptotic cores were also present in all conditions (data not shown). In quantitative analysis of apoptotic nuclei, the range of apoptosis was generally, though not entirely, the fitting counterpart to percent viability in live/dead analysis. Significant differences were observed mainly between free control and bioprinted NICC. On day 1 after bioprinting, the control NICC contained a significantly lower percentage of apoptotic cells than those bioprinted in BSA- and PL-algMC. On day 21 on the other hand, apoptotic nuclei in free control NICC were lower than in NICC bioprinted in PBS-, BSA- and PL-algMC. NICC bioprinted in FFP-algMC were comparable to free control NICC at all time points. In a comparison between the bioinks, apoptosis was significantly reduced in NICC bioprinted in FFP-algMC on day 7 with no significant differences at other time points. Analogous to previous results (Figure S1) on day 7 after bioprinting, control islets were notably larger than the bioprinted ones, but islet size was not further influenced by the type of bioink.

As neonatal islets are known for a high rate of proliferation, especially compared to adult islets, NICC were additionally stained for proliferating nuclei (Figure 3C,E). In all analysed NICC, EdU-positive nuclei were detected over the entire time of observation, with a mean area of 10% on day 1 after bioprinting followed by a stark drop in proliferation towards day 21 in all groups. Proliferation was generally comparable between the groups, with no significant differences between the free control and the PBS-, BSA-, and PL-bioinks. NICC bioprinted in FFP-algMC on the other hand showed a significantly higher proliferation than all other groups on day 1 after bioprinting. In addition, in FFP-algMC, proliferation was reduced over time resulting in an only slightly higher proliferation on day 7 and no difference between the groups on day 21 after bioprinting.

Furthermore, as full scaffolds instead of cryosections were imaged, these results again reinforce the qualitative impression that the overall morphology of the islets is not impacted by incorporation into the highly viscous bioinks, nor by the extrusion-based printing process. Instead, visible is a distinct tendency of free control NICC to start to disintegrate at later time points, exemplarily indicated by the upper right image (free control, d21) in Figure 3E.

### 3.3. Functionality of Bioprinted NICC

Despite their comparatively low number of β-cells and the need for maturation before NICC are able to normalise blood glucose levels in vivo [7,15,16,17], they are able to produce insulin and can show a functional response in vitro early on [12,46]. In the present work, we determined whether they also show a functional response after bioprinting. Functionality was assessed by analysing the production and localisation of different pancreatic hormones, the number of insulin-positive cells, and the release of insulin in response to stimulation with glucose.

**Figure 4:**
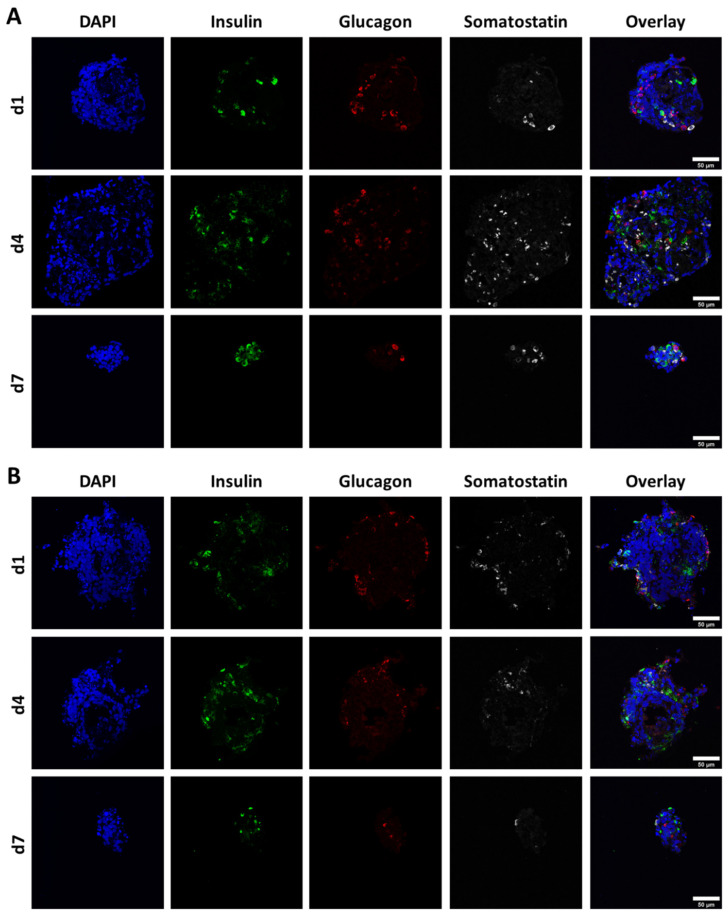
Representative images of NICC immunofluorescently stained for nuclei (DAPI), insulin, glucagon, and somatostatin. (**A**) Free control NICC; (**B**) NICC in bioprinted PBS-algMC scaffolds. Samples were incubated for 1, 4, or 7 d under cell culture conditions. Scale bars = 50 µm.

For production and localisation of pancreatic hormones, exemplary cryosections of free control and bioprinted NICC in non-supplemented algMC (PBS-algMC) fixed on day 1, 4, and 7 after bioprinting were stained for insulin, glucagon, somatostatin, and nuclei (Figure 4). All three hormones could be detected in all analysed NICC of both control and bioprinted samples throughout the whole time of observation, yet were only prevalent in small amounts and a low number of cells within the islet clusters. This was mostly independent of islet size or shape, and cells positive for insulin, glucagon, and somatostatin were distributed randomly within the NICC.

**Figure 5:**
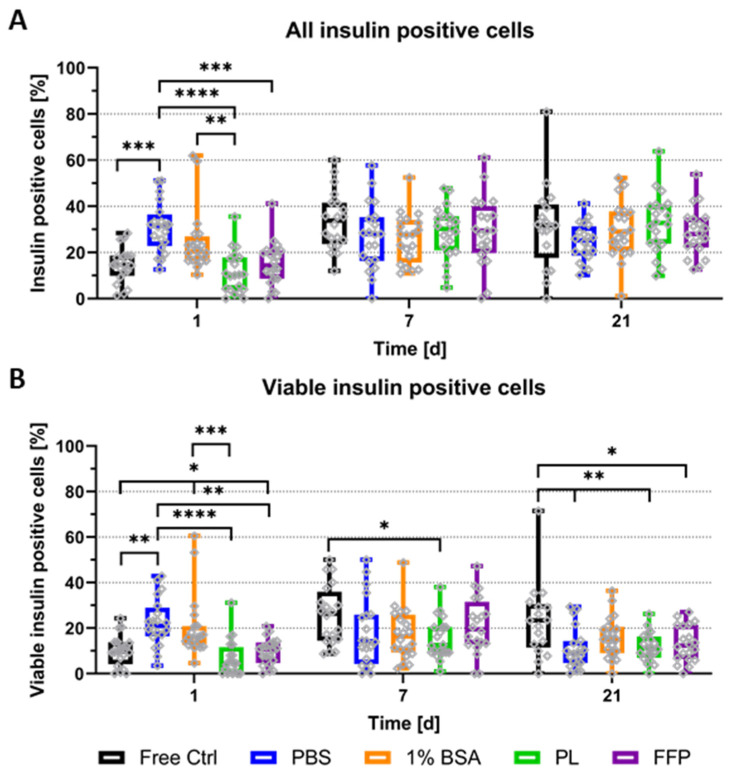
Quantitative analysis of insulin-producing cells in NICC. Analysed groups are free control NICC in suspension culture and NICC bioprinted in PBS-algMC, BSA-algMC, PL-algMC, and FFP-algMC. (**A**) Percent insulin-positive of all cells per islet cluster, including double-positive cells for insulin and TUNEL; (**B**) Percent viable insulin-positive of all cells per islet cluster, excluding double-positive cells for insulin and TUNEL. Mean ± SD, *n* = 1, 20 NICC. Grey dots in all graphs indicate individual islets, significances indicate * *p* < 0.05, ** *p* < 0.01, *** *p* < 0.001, **** *p* < 0.0001.

For a more in-depth analysis of specifically the presence and amount of insulin-containing cells, cryosections of control NICC and NICC bioprinted in (supplemented) algMC gels were stained for insulin, nuclei, and apoptotic nuclei. As graphically depicted in Figure S4, the nuclei were sorted into the categories viable insulin-negative (DAPI), apoptotic insulin-negative (TUNEL), viable insulin-positive (DAPI and insulin), and apoptotic insulin-positive (TUNEL and insulin) via manual counting and percentages of insulin-containing cells were calculated according to the complete cell number. Figure 5A depicts the percentage of all insulin-producing cells (viable and apoptotic insulin-positive of total), whereas in Figure 5B only viable insulin-containing cells (excluding those double-positive for insulin and TUNEL) are plotted. The vast majority of analysed NICC did contain insulin-positive cells in a wide distribution with some clusters containing almost no, others up to 60% insulin-positive cells. On day 1 after bioprinting, NICC in un-supplemented PBS-algMC, and partially also in BSA-algMC, contained significantly more insulin-positive cells than those in the free control, PL-, and FFP-algMC. At later time points all groups were comparable.

Differences between the conditions are more clearly apparent when cells, those double-positive for TUNEL and insulin, are excluded, and only viable insulin-containing cells are considered (Figure 5B). Viable insulin-positive cells increased over time in free control NICC. Compared to bioprinted NICC, their number was comparable to PL- and FFP-algMC, smaller by trend than BSA-algMC, and significantly smaller than PBS-algMC on day 1. From day 7 onwards on the other hand, free control NICC contained more viable insulin-positive cells than those in bioprinted scaffolds, partially by trend, partially significantly more. This is only the case when apoptotic insulin-positive cells are excluded in accordance with an overall higher amount of apoptotic nuclei (Figure 3B) between free control and bioprinted NICC on day 21. While the overall amount of insulin-positive cells is similar, a higher amount of those are undergoing apoptosis in bioprinted NICC than in free control. If the bioinks are compared amongst themselves, percentage of viable insulin-positive cells in PBS-algMC is significantly highest on day 1, but lowest by trend on day 21. Furthermore, on day 1, NICC in BSA-algMC contained significantly more insulin-positive cells than those in FFP-algMC and more by trend than those in PL-algMC. At later time points, no differences between the bioinks were observed.

**Figure 6:**
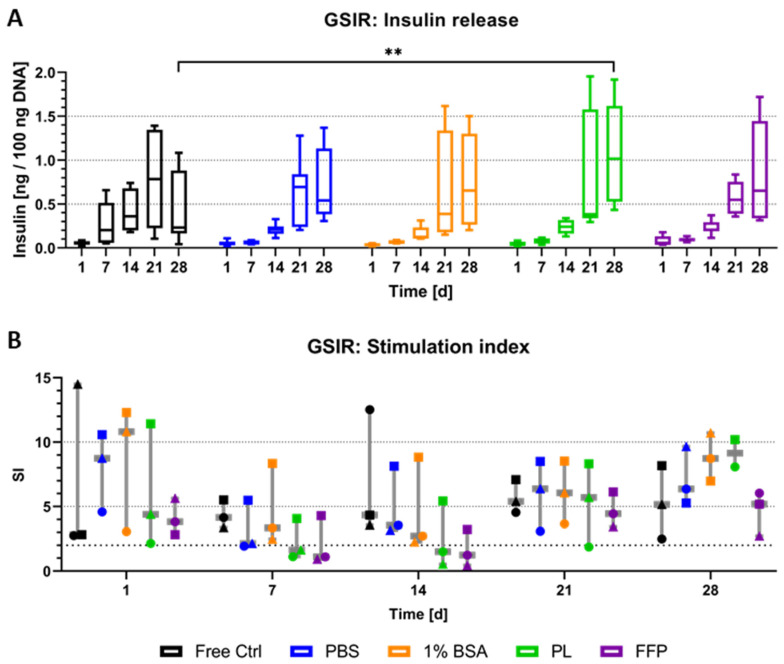
Glucose-stimulated insulin release of NICC. Analysed groups are free control NICC in suspension culture and NICC bioprinted in PBS-algMC, BSA-algMC, PL-algMC, and FFP-algMC. (**A**) Amount of insulin released in response to stimulation with high (16.4 mM) glucose normalised to 100 ng DNA. Mean ± SD, *n* = 9 replicate values from 3 replicate exp., significances indicate ** *p* < 0.01. (**B**) Stimulation index as ratio of insulin released during stimulation with high (16.4 mM) and low (3.3 mM) glucose. Mean ± SD, *n* = 3 exp., 3 replicates each, the 3 different experiments are distinguished by different symbols.

The detection of insulin-positive cells in the majority of NICC analysed was followed up by the investigation of a functional reaction to glucose stimulation (Figure 6). In preliminary experiments with free control and NICC bioprinted in PBS-algMC, we had observed the release of insulin but had not been able to observe a functional reaction to glucose stimulation. In those experiments, the amount of insulin released in reaction to high glucose and to low glucose had been highly similar, visible as a stimulation index (SI) of below two in both conditions over a week. As a general rule, neonatal islets need to mature before they become functional, but it is possible to enhance insulin release by exposure to glucagon-like peptide-1 (GLP-1) [47]. Therefore, in the present study, liraglutide, a long-lasting GLP-1 analogue, was added during high-glucose stimulation. Free control and bioprinted NICC were exposed to low and high glucose and insulin release was measured by ELISA. Figure 6A depicts the absolute amount of insulin released in response to high glucose stimulation after normalisation to the DNA content. Insulin release increased strongly over time, starting on day 14, with a much stronger increase at day 21 after bioprinting. While this was observed in all analysed groups, with high standard deviations at later time points, there was a definitive trend for increase in absolute amount released from bioprinted NICC than from free control NICC on day 28. This trend was not reflected in significant differences though, except in case of PL-algMC.

The stimulation index (i.e., the ratio calculated from insulin release in high, divided by insulin release in low-glucose stimulation) is depicted in Figure 6B, the bold dotted line indicates an SI of greater than two, which is generally considered a functional response. Each data point reflects the mean of an entire experimental data set, i.e., three replicate samples per group per time point. Overall NICC showed a functional response in all analysed groups at all time points, in at least one, but most often all three of the repeat experiments. Generally speaking, differences between the repeat experiments (i.e., differences between the isolations) were larger than those between the experimental groups, which resulted in no significant differences between any of the conditions. Furthermore, in the majority of cases, though not always, NICC within one experiment (denoted by similar symbols) reacted similarly. This is especially clearly illustrated for PBS-, BSA-, and PL-algMC on day 21, whereby the mean SI of the first experiment ranged from 2–4, the second one from 8–9, and the third from 6–7, independent of the bioink. In a direct comparison between the bioinks, PBS-, and BSA-algMC resulted in a slightly higher SI by trend than the use of PL-, and FFP-algMC.

## 4. Discussion

The blend of 3% alginate and 9% methylcellulose (algMC) used in this study has been shown to be compatible with a variety of cell types in the past [27,43,48,49,50]. With this highly viscous blend, it is possible to (bio)print scaffolds that retain their shape until crosslinking yet stay in pressure ranges that do not negatively impact single cells via shear stress [51]. Furthermore, extruding pancreatic islets in algMC through an 840 µm nozzle with 30–50 kPa we could show no impact on the viability in our previous work [31]. Analogous to these results, for the bioprinting of NICC in the present study with similar conditions, percent viability and apoptosis were generally comparable between free control and bioprinted NICC, indicating no impact of shear stress during incorporation and extrusion printing.

The distribution of apoptotic cells in the islets is generally comparable between free control and bioprinted NICC. The sole difference being that in a low number of bioprinted NICC, but not in free control NICC, an outer ring of apoptotic nuclei surrounding a core of healthy cells was observed. While this might be attributed to shear stress, it first became apparent on day 4 after printing and as the distribution of apoptotic nuclei is otherwise comparable between the control and bioprinted NICC, it is likely that free NICC also contain apoptotic cells at the borders but that those can detach from the cluster in suspension culture. Additionally, control, as well as bioprinted NICC, contained large apoptotic areas, apoptotic cores, and empty areas. This is in accordance with the eventual disintegration of dying cells and has been observed by other groups in the past, especially for central necrotic areas after 7 days of culture [52,53]. Overall, the viability observed in the present study with a mean of 60–70% is slightly lower than what is reported by other groups, where viability in the suspension culture ranged from 80–90% after 8 days [54,55,56], 75% after 7 days [57], and 50% after 20 days [53]. As NICC were kept in culture for 7 days before bioprinting in the present study, these time points are approximately comparable to the day of printing and day 12 after printing. Concerning the long-time culture of encapsulated NICC, the time frame observed herein is in accordance with the literature, as the survival of NICC in alginate-based capsules in vitro has been reported for at least 5 weeks as measured by oxygen consumption [58].

Taken together, the viability data (Figure 2) show that the algMC blend adequately maintains viability and morphology of NICC equivalent to suspension culture. However, literature suggests that supplementation with proteins such as albumin [39] or protein-growth factor mixtures such as PRP [40,59] can lead to an increase in viability, β-cell proliferation, and insulin secretion in adult murine pancreatic islets. It is therefore feasible, that the addition of such supplements might also increase viability in bioprinted NICC. Specifically for the variants tested for NICC here, it was reported that supplementation with human serum albumin improved the stability of alginate microcapsules and increased viability and insulin secretion of encapsulated murine islets over 3 weeks of culture [39], and after implantation, islets in such microcapsules were able to restore normoglycaemia in mice [60]. Furthermore, Bertera et al. could show improved blood glucose control in mice if BSA was added during transplantation [61]. A highly supportive effect has also been achieved with more complex supplements consisting of a physiological mixture of various proteins and growth factors such as PRP. In the majority of studies, PRP was used as a liquid supplement and added to islets in in vitro culture or, if used in vivo, was directly injected into diabetic animals and the effect on the diabetic pancreas was observed. For rat islets in vitro, this resulted in an increased viability, as well as an increased insulin content and release with no reported SI [59]. In vivo, when diabetes was induced in rats, the injection of PRP led to an increase in the number of β-cells, increased insulin secretion, improved blood-glucose control, and reduced oxidative stress [40,41,59,62]. A positive effect on the number of β-cells was also observed when PRP was added during the differentiation of induced pluripotent stem cells [63]. When PRP was used in combination with biomaterials, specifically alginate and decellularized pancreatic extracellular matrix (dECM), this resulted in an improved viability, a higher insulin gene expression, and a higher insulin release from β-cell lines [64,65]. In the present study, we used platelet lysate produced from PRP. For FFP, to the best of the authors’ knowledge, no studies with pancreatic islets have been published, it was deemed a possible candidate due to its positive effect on other cell types, especially in combination with algMC though. Specifically, FFP was used to create a human skin model with keratinocytes and fibroblasts [66], and when used to supplement algMC, stimulated the proliferation and differentiation of bioprinted mesenchymal stromal cells [67] and increased proliferation and albumin production (an indicator for functionality) in a hepatocyte cell line [48].

Furthermore, in a comparison of free control NICC and NICC bioprinted in supplemented bioinks (Figure 3), no major differences in viability were apparent. In a comparison between the different bioinks, few significant differences were observed, though there is a definitive trend for higher viability after supplementation with PL, but mainly FFP, which is reflected in a trend by a higher viability from day 14 onwards for both. Furthermore, only supplementation with FFP resulted in fewer apoptotic nuclei from day 7 onwards and a higher proliferation until day 7. In contrast to this, in vitro other groups observed a definitive increase in the viability of murine islets after the addition of serum albumin to alginate [39], an increased viability of β-cell lines when PRP was added to alginate [64] or to pancreatic dECM [65], and an increased viability of murine islets when PRP was added to the culture medium [59]. Of these, only the study by Schneider et al. with albumin-supplemented alginate analysed a time frame of more than one week [39], whereas the effect seen in the in vitro studies with PRP [59,64,65] was reported for a maximum of 5 days.

In a more detailed comparison of the bioinks, BSA-algMC was supplemented with 10 mg/mL albumin, whereas PL [68,69] and FFP [70] have been reported to contain albumin in a concentration of 30–40 mg/mL. In addition, both contain >2 mg/mL fibrinogen and various growth factors, though those are present in higher concentrations in PL than in FFP because in the latter case only some, not all, platelets are lysed [67,68,69]. While the supplements were present during crosslinking and could partially be trapped within the alginate network, none of them are bound to the matrix and can therefore be released over time. Specifically for BSA, a quick release from alginate beads within hours has not only been reported [71] but also observed in our lab (unpublished data). For growth factors and un-crosslinked fibrinogen a somewhat slower release can be expected [72], though other groups have also reported “no detectable leakage from alginate capsules” [73] and our group observed a positive effect on the proliferation and viability of MSC over 4 weeks [67]. The quick release of supporting supplements could explain a temporary effect seen in the short-term studies with PRP and not present at the time points analysed in the present work, yet Schneider et al. observed an increased viability over 3 weeks if 1% (10 mg/mL) human serum albumin was added to alginate. On the other hand, they used Ba^2+^ ions for crosslinking, which resulted in a very dense polymeric network, so the effect of the albumin might have been more visible than in the microporous [27,43] algMC used here.

The apoptosis of islet cells, usually present if islets are cultured in vitro [74], is more pronounced in NICC as they undergo a strong remodelling/maturation process during which their cell composition can change from 17 to 94% β-cells [54]. The increase in the proportion of β-cells during maturation is partially due to the death of exocrine cells [54], partially due to the inherent capacity for the expansion of neonatal β-cells that has been described by many researchers [53,54]. For example, Nielsen et al. [52] analysed proliferation via staining for the proliferation marker Ki67, measured via fluorescence-activated cell sorting (FACS), and observed a proliferation rate of 35% directly after isolation (a time point not analysed here) and 15% on day 14 of the culture (equivalent to day 7 after bioprinting). Overall proliferation in the present study, analysed via exposure to EdU for 24 h, was comparatively low, with a mean of 5–10% proliferating cells in the first week after bioprinting, which dropped to below 2% on day 21. This was also observed in the free control though, and can therefore not be attributed to the bioprinting but is more likely an artefact of batch variation. While other research has indicated that especially PRP can lead to the increased proliferation of pancreatic (β-)cells in vivo [40,62], we detected a positive influence of FFP-supplementation at early time points but not of PL-supplementation, despite mostly similar compositions. On the other hand, during in vivo studies, PRP was injected twice weekly and proliferation was seen in adult rats [40,59], whereas supplements were released from bioinks over time and not replenished. Possibly a beneficial effect on NICC could also be observed if supplements were (additionally) added to the culture medium.

When NICC were analysed for functionality in the present study, samples were also exemplarily stained for glucagon and somatostatin between day 1 and 7 after bioprinting, but focus lay on analysing insulin-positive cells over 21 days after bioprinting (28 days after isolation). For this timeframe, NICC have been reported to contain 14–24% insulin-positive cells after 1 week of culture [12,54,75], or 20–25% after 14 days [52,57]. At the corresponding time points, observations in the present study (day 1 and day 7 after bioprinting), with 5–25% healthy or 10–30% total insulin-containing cells, are in line with those results. On the other hand, the further increase in β-cell number over time, observed mainly after transplantation and seen for non-encapsulated [54], as well as alginate-encapsulated NICC [76], was not observed here. It is plausible that NICC mature differently in vitro than in vivo though, for example, Nielsen et al. observed an increase over time in vitro, with a maximum of 20% insulin-positive cells on day 14 of culture, followed by a decrease towards day 18 [52].

In light of the low number of β-cells and in accordance with the literature [12], 50,100 free NICC per replicate, and a density of up to 20,000 IEQ per gram ink were used for stimulation experiments in the present study (Figure 6). The purpose of pancreatic β-cells is the release of insulin in response to elevated glucose levels—a functional response that only develops after birth, as foetal islets do not react to stimulation. In neonates, the functional response develops over a period of several weeks, which is reflected in transplantation studies where NICC have been shown to regulate blood-glucose levels only after several weeks in vivo [12,53,54,57,77]. In accordance with this, in preliminary experiments we could not observe a functional response, neither in bioprinted nor in free control NICC (unpublished data). However, even in the immature state, NICC can be driven to insulin secretion in response to high glucose in combination through agents increasing the levels of cyclic adenosine-monophosphate (cAMP), such as theophylline [12,47,78], forskolin, and arginine, or with GLP-1 analogues [47] such as the liraglutide employed in the present study. Encapsulation in alginate-based matrices does not hamper this maturation in vivo [76] nor in vitro [79] and is also clearly represented in an increased insulin release starting from day 14 after bioprinting (day 21 after isolation) in the present study. Increased insulin release indicating maturation was observed in all groups and in comparable amounts between free control and bioprinted NICC (Figure 6A). However, in contrast to reports by other groups, in the present study, supplementation of bioinks did not further increase insulin secretion in a notable fashion. On the other hand, only a low number of studies with serum albumin have been performed [39] and when PRP was used it was either a short time of observation [59,64,65], or repeated supplementation [40,41].

The SI, the ratio between insulin released during high and low glucose stimulation illustrates functionality in all groups over a duration of 4 weeks in vitro, even with a slight trend for a higher SI in bioprinted than in free control samples on day 28 after bioprinting. The mean SI in the free control lay between 5 and 7 between day 7 and day 35 after isolation (i.e., day 1–day 28 after bioprinting). With the addition of theophylline, the SI has been reported as 17 after 1 week [80], as 2.7 [81], as well as 40 [12] after 10 days; with the addition of arginine as a 4–5-fold increase in high glucose on day 9 [52]. For NICC encapsulated in alginate, Park et al. [56] reported an SI of two without cAMP-increasing agents after 2 and 4 weeks, and Mouré et al. [82] using theophylline reported an SI of three after 3 days and an SI of 13 calculated as a mean of 2 weeks. Tatarkiewicz et al. [81] on the other hand reported non-functionality (SI = 1.3) even with theophylline, after 10 days of culture. In line with the literature, the mean SI of bioprinted NICC in the present study lay between 3 and 8 in un-supplemented PBS-algMC, between 5 and 9 in BSA-algMC, between 2 and 9 in PL-algMC, and between 1.8 and 5 in FFP-algMC. Similarly to insulin release, no benefit of supplementation on functionality was observed. While in the above-mentioned in vivo studies functionality could be shown through increased blood-glucose control, there are few studies observing the influence of such supplements in vitro and reporting the SI. Furthermore, none of the studies exploring these supplements before worked with NICC, but rather with adult murine islets. For example, Duruksu et al. described an increased insulin secretion but no effect on the SI for NICC encapsulated in alginate beads and supplemented with PRP [64]. In light of this it might be that the effect of especially PRP is mainly visible in vivo, but does not lead to significant changes in vitro. Nevertheless, the data presented here does indicate that NICC can be bioprinted into alginate-based scaffolds while retaining their functionality comparably to free control for at least 4 weeks in culture.

The present work only concerns itself with The general feasibility of using 3D bioprinting in combination with NICC and the analysis of the bioprinted scaffolds in vitro, but can be seen as a first step on the way to applying this system also for the transplantation of islets. In previous studies detailing the transplantation of encapsulated islets, alginate has been shown to be strongly immunoprotective, as illustrated strikingly by the work of Elliott et al. published in 2007, in which they transplanted alginate-encapsulated porcine islets into a human patient and while the transplant lost function over time they did not report a notable immune response for over almost 10 years in vivo [17]. In our work we have used alginate in combination with methylcellulose, which, while not as extensively researched as alginate, has also been reported as biocompatible [83,84], and the cellulose derivative hydroxypropyl methylcellulose has been used as a component of a degradable gel for the transplantation of islets in rats [85]. While the algMC used here was analysed thoroughly in vitro, it has not yet been used in vivo, and it cannot be excluded that the presence of methylcellulose, which changes the microstructure of the alginate network [27,43], might result in slightly lower immunoprotective capacities than has been observed for plain alginate. As the immunoprotective capacities cannot be inferred from studies in vitro, it is imperative that the results presented here are followed up by an in vivo study analysing the immune response to cell-free and cell-containing scaffolds as well as the functionality of NICC bioprinted in algMC.

## 5. Conclusions

With the data presented here we could show the successful bioprinting of neonatal porcine islet-like cell clusters in alginate-methylcellulose bioinks. The bioprinted macroporous scaffolds remained stable over the entire time of observation, and encapsulated NICC demonstrated a high viability and functionality over 4 weeks in culture. The material was additionally supplemented with bovine serum albumin or the human blood plasma derivatives platelet lysate and fresh frozen plasma, which did not significantly affect viability nor functionality.

## Data Availability

The full datasets generated for this study are available from the corresponding author on request.

## Supplementary Materials

The following supporting information can be downloaded at: https://www.mdpi.com/article/10.3390/biomedicines10061420/s1, Figure S1: Size distribution of NICC in suspension culture and bioprinted in algMC scaffolds. Figure S2: Macroscopic view of the distribution, insulin content (DTZ), and metabolic activity (MTT) of bioprinted NICC in (supplemented) bioinks. Figure S3: Size distribution of NICC in suspension culture and bioprinted in (supplemented) algMC scaffolds. Figure S4: Exemplary depiction of a cryosection used for quantitative analysis of insulin-containing cells within NICC.
