# Supplementary material for: Viability and Functionality of Neonatal Porcine Islet-like Cell Clusters Bioprinted in Alginate-Based Bioinks
Source: Biomedicines. 2022 Jun 15;10(6):1420. doi: 10.3390/biomedicines10061420 (PMC9220255; doi:10.3390/biomedicines10061420)
Supplement: Supplementary file 1 [file biomedicines-10-01420-s001.zip › biomedicines-1760694-supplementary.pdf]

Supplementary information

For

Viability and functionality of neonatal porcine islet-like cell clusters bioprinted in alginate-based hydrogel blends

Sarah Duin[1*], Shreya Bhandarkar[1], Susann Lehmann[2], Elisabeth Kemter[3], Eckhard Wolf[3], Michael Gelinsky[1], Barbara Ludwig[2,4], Anja Lode[1]

[1] Centre for Translational Bone, Joint and Soft Tissue Research, University Hospital *Carl Gustav Carus* and Faculty of Medicine of Technische Universität Dresden, Germany

[2] Paul Langerhans Institute Dresden of Helmholtz Centre Munich at University Hospital Carl Gustav Carus, Technische Universität Dresden and German Centre for Diabetes Research (DZD), Dresden, Germany

[3] Chair for Molecular Animal Breeding and Biotechnology, Gene Center, and Center for Innovative Medical models (CiMM), Ludwig-Maximilians University Munich, München, and German Centre for Diabetes Research (DZD), Neuherberg, Germany

[4] Medical Clinic III, University Hospital Carl Gustav Carus, Technische Universität Dresden, Germany

* Corresponding author: Sarah Duin, telephone: +49 351 458-16692, fax number: +49 351 458-7210, email address: sarah.duin@tu-dresden.de

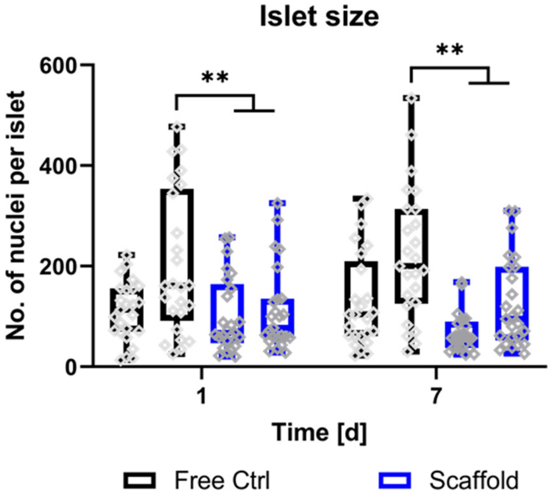

Supplementary Figure S1: Size distribution of NICC in suspension culture and bioprinted in algMC scaffolds determined by means of counting nuclei in DAPI stained 2D cross-sections. Mean ± SD, n=2 experiments, 25 islets each, ***p*<0.01. Repeat experiments depicted as neighbouring box plots, grey dots indicate individual islets.

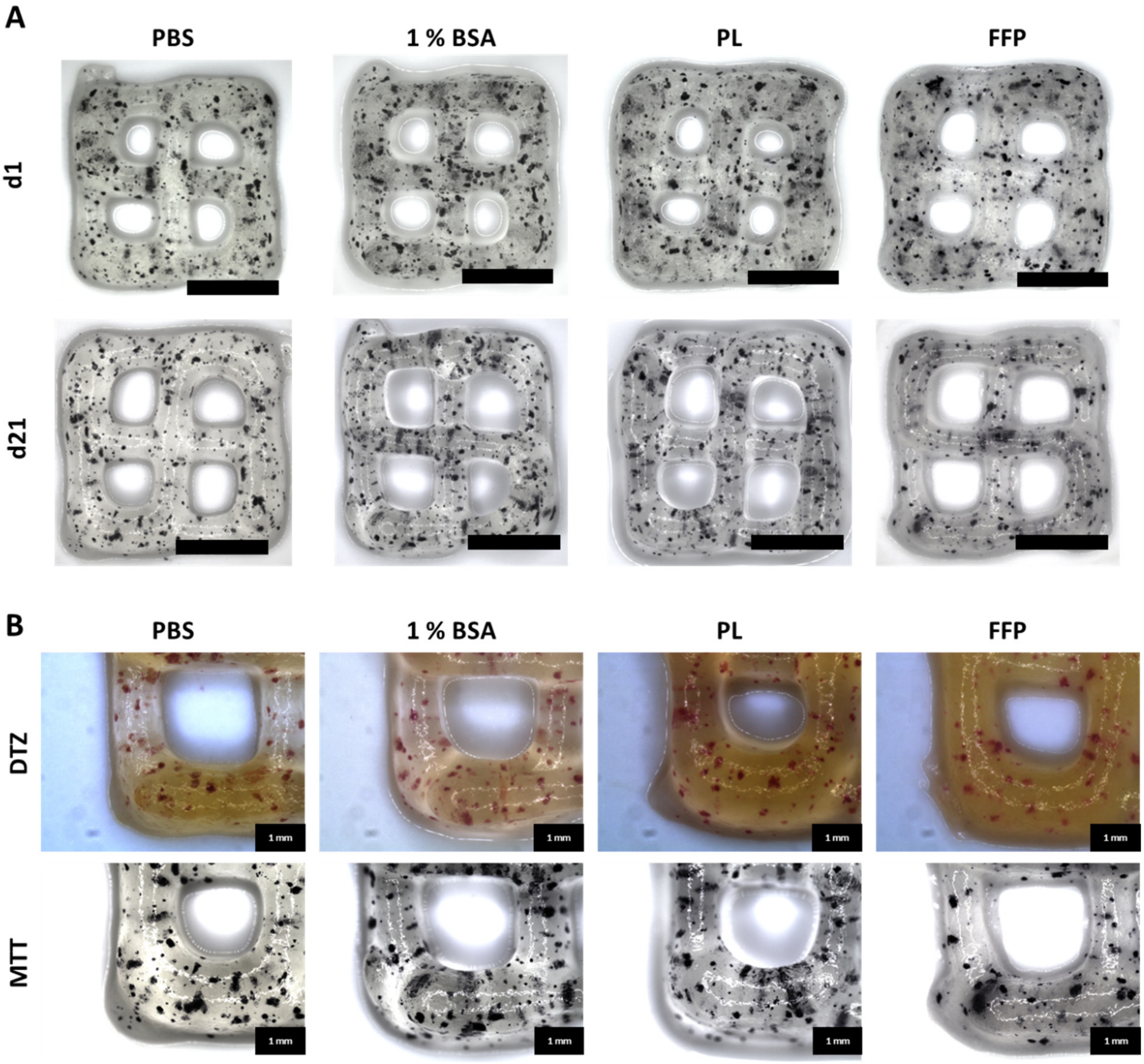

Supplementary Figure S2: Macroscopic view of the distribution, insulin content (DTZ), and metabolic activity (MTT) of bioprinted NICC in (supplemented) bioinks. Depicted are NICC bioprinted in PBS-algMC, BSA-algMC, PL-algMC, and FFP-algMC on d21 after bioprinting. (a) Representative images of the entire scaffold on day 1 and day 21 of incubation, showing form-stability. NICC stained with MTT, scale bars = 2 mm. (b) Magnified view of one corner of the scaffolds, NICC stained with DTZ or MTT, scale bars = 1 mm**.**

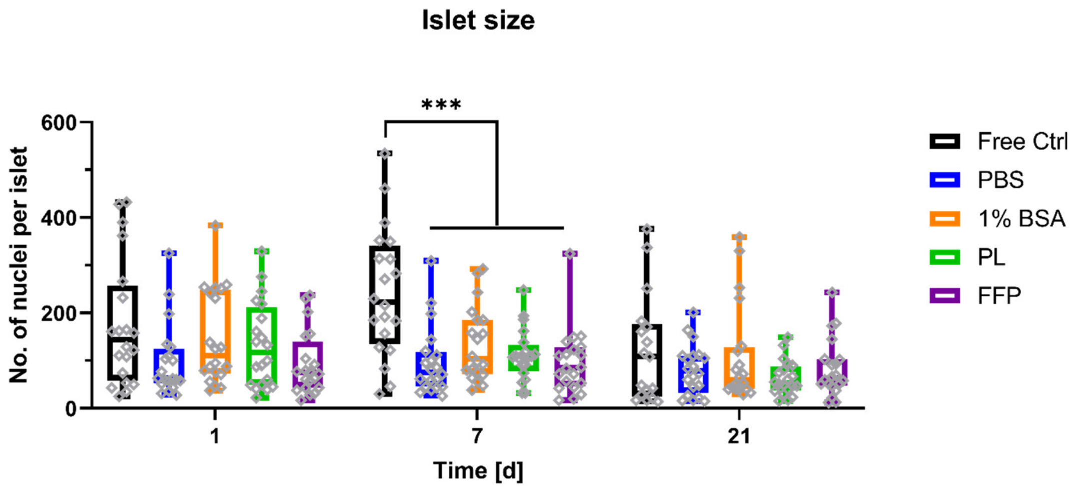

Supplementary Figure S3: Size distribution of NICC in suspension culture and bioprinted in (supplemented) algMC scaffolds determined by means of counting nuclei in DAPI stained 2D cross-sections. Mean ± SD, n=1 experiment, 20 islets, ***p<0.001, grey dots indicate individual islets.

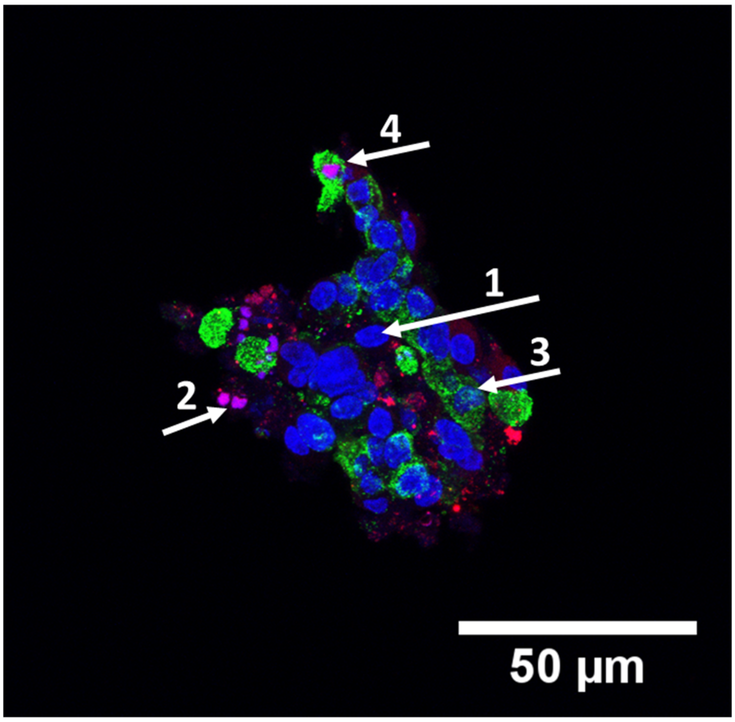

Supplementary Figure S4: Exemplary depiction of a cryosection used for quantitative analysis of insulin-containing cells within NICC. Indicated by numbered arrows, the different states that were considered are viable (1), apoptotic (2), viable insulin-containing (3), and apoptotic insulin-containing (4).
